# Role of Immune Cells in Mediating the Effect of Hypothyroidism on Idiopathic Pulmonary Fibrosis

**DOI:** 10.1111/crj.70111

**Published:** 2025-07-10

**Authors:** Zhengling Liu, Chengkun Kou, Xiaobo Chen, Jing Yang, Huan Zhu, Yongning Jiao, Dongyan Zhang, Wencui Zhang, Liang Li

**Affiliations:** ^1^ Department of Respiratory Medicine Department Gansu Province Central Hospital & Gansu Provincial Maternity and Child Care Hospital Lanzhou Gansu China; ^2^ The Second Clinical Medical School, Lanzhou University Lanzhou Gansu China

**Keywords:** hypothyroidism, idiopathic pulmonary fibrosis, immune cell, Mendelian randomization

## Abstract

**Introduction:**

Idiopathic pulmonary fibrosis (IPF) leads to irreversible scarring of lung tissue, resulting in deteriorating respiratory function, particularly in older adults. We aimed to explore the causative link between hypothyroidism and IPF, particularly focusing on immune cell phenotypes as mediating factors.

**Methods:**

A two‐sample Mendelian randomization (MR) approach was utilized to investigate the influence of hypothyroidism on IPF and the role of 731 distinct immune cell phenotypes as mediators. The mediating effects were quantified using the coefficient product method. Various sensitivity analyses, including Cochran's *Q* test for heterogeneity, MR–Egger for pleiotropy, and the “leave‐one‐out” method, were conducted to verify the robustness of single‐nucleotide polymorphism–derived casual estimates. Statistical analyses were carried out using the R software (Version 4.3.1).

**Results:**

Hypothyroidism was significantly associated with increased IPF risk (odds ratio [OR] = 1.13, 95% confidence interval [CI] = 1.06–1.21, *p* = 1.34 × 10^−4^). Of the 36 immune cell phenotypes associated with IPF, those related to the mean fluorescence intensity of B cells were the most prevalent. Mediation analysis showed that CD19 on IgD− CD27− accounted for approximately 3.68% of the effect of hypothyroidism on IPF, whereas herpesvirus entry mediator (HVEM) on T cells accounted for approximately 3.83% of this effect.

**Conclusion:**

We identified a marked association between hypothyroidism and IPF. Specific immune cell phenotypes may partially mediate this relationship, although the observed effect sizes were modest. Further research is needed to validate these results in diverse populations and larger clinical trials.

## Graphical abstract

- This study determined the effect of hypothyroidism on IPF using a two‐sample MR approach.
- Mediating effects of immune cells were assessed using the coefficient product method.
- Hypothyroidism significantly increased the risk of developing IPF.
- CD19 on IgD− CD27− and HVEM on T cells were identified as critical mediators.
- Hypothyroidism likely contributes to IPF pathogenesis through immune dysregulation.

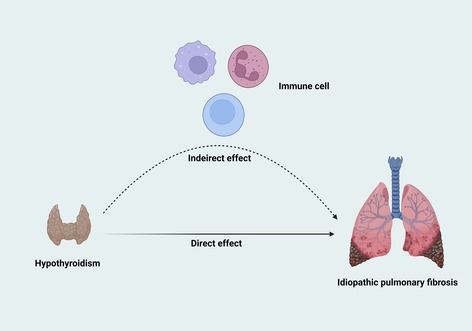

## 1 Introduction

Idiopathic pulmonary fibrosis (IPF) is a prevalent form of interstitial lung disease, accounting for approximately one‐third of all cases within this category [1]. IPF is defined as a slowly advancing fibrotic interstitial pneumonia of uncertain etiology, characterized by the excessive accumulation of extracellular matrix, with collagen being the primary component [2]. This condition primarily affects older adults, manifesting as progressive dyspnea and a gradual reduction in lung function, ultimately leading to respiratory failure [3]. Its prognosis remains poor, with elevated mortality and a median survival duration of less than 5 years [4].

Previous research indicates that hypothyroidism is a prevalent comorbidity among patients with IPF [4]. Meta‐analysis evidence suggested that hypothyroidism may contribute to chronic fibrosis in various organs, including the heart, liver, and lungs [5]. A retrospective study indicated that the occurrence of hypothyroidism among individuals with IPF varies between 13% and 28% [4], thereby significantly exceeding the general population rate of 1%–9% [6]. Recent studies utilizing Mendelian randomization (MR) have identified hypothyroidism as a risk factor for IPF [7].

Immune cells are major target cells for thyroid hormones [8]. Previous research revealed that patients with hypothyroidism exhibit decreased levels of peripheral blood CD8+ T cells alongside a higher CD4/CD8 ratio [9]. Inflammatory responses and immune system dysregulation are key pathophysiological features of IPF [10], and several studies have confirmed the relationship between immune cells and IPF [11]. Notably, a prior MR study revealed that CD39 on CD39+ CD8+ T cells conferred a protective effect against IPF [12]. Regulatory T, CD8 + T, and natural killer (NK) cells are also critical contributors to IPF pathogenesis [13]. These findings have highlighted immune cells as potential mediators between hypothyroidism and IPF.

MR analysis is an analytical approach that utilizes genetic variants as instrumental variables (IVs) to establish causal links between a risk factor and its associated outcome in observational research. To fill these gaps in the literature, in the present study, we aimed to explore the mechanism by which immune cells mediate the effect of hypothyroidism on IPF using MR, further clarifying the mechanisms through which hypothyroidism affects IPF and offering new insights for its treatment.

## 2 Methods

### 2.1 Study Design

A two‐sample MR analysis was conducted to assess the causal relationship between hypothyroidism and IPF and to investigate the mediating role of immune cells. MR is an epidemiological method that employs genetic variants, typically single‐nucleotide polymorphisms (SNPs), as IVs to assess potential causal relationships between an exposure (e.g., hypothyroidism) and an outcome (e.g., interstitial lung disease). MR relies on three key assumptions: (1) the selected genetic variants, proposed as IVs, must be strongly associated with the risk factor being studied, (2) these genetic variants should not be correlated with any potential confounding factors, and (3) the chosen genetic variants should influence the outcome risk exclusively through the risk factor of interest, rather than through any alternative pathways [14].

**FIGURE 1:**
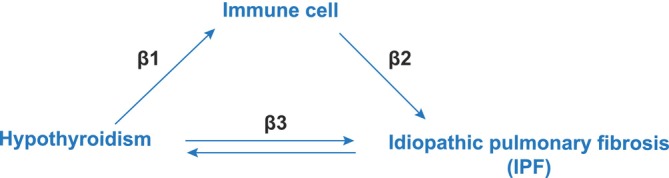
Overview of the present study design.

Additionally, the mediating effect of immune cells in this relationship was evaluated (Figure 1) using the coefficient product method [15]. This involved calculating the effect of hypothyroidism on immune cells (β1) and their subsequent effect on IPF (β2). The overall mediatory influence was quantified as the fraction of the overall effect of hypothyroidism on IPF (β3), modulated by immune cells, determined using the formula (β1 × β2)/β3 [16]. This method allowed us to delineate the extent to which immune cells serve as intermediaries in the hypothyroidism–IPF relationship, providing a new understanding of the fundamental biological processes.

### 2.2 Data Source

#### 2.2.1 Genome‐Wide Association Studies (GWAS) Data for Hypothyroidism

Genetic variants associated with hypothyroidism were obtained from the UK Biobank (UKB) database on April 10, 2024. The UKB study is designed as a long‐term cohort analysis that provides extensive genomic and phenotypic information. Approximately 500 000 participants contributed to a rich dataset pertaining to phenotype and health metrics, encompassing lifestyle metrics, along with biomarkers derived from blood and urine samples, and body and brain imaging [17]. The GWAS analysis included 20 563 hypothyroidism cases and 399 910 controls, limited to individuals of European ancestry (https://pan.ukbb.broadinstitute.org/).

#### 2.2.2 GWAS Data for IPF

Genetic markers linked to hypothyroidism were obtained through the FinnGen Consortium, which collects and analyzes genomic information derived from 500 000 Finnish biobank participants. The GWAS summary statistics included 2401 IPF cases and 448 636 controls (https://r11.finngen.fi/).

#### 2.2.3 GWAS Data for Immune Cells

Genetic markers linked to immune cells were obtained from the GWAS catalog (GCST0001391 to GCST0002121), based on data from 3757 participants of European descent across various cohorts. A total of 731 immune cells were analyzed [18], including absolute cell counts (AC), median fluorescence intensity (MFI), morphological parameters, and relative cell counts. The study focused on diverse immune cells such as mature B cells, conventional dendritic cells (cDCs), T cells, monocytes, and myeloid cells and included detailed panels for TBNK cells (T cells, B cells, and NK cells) and Treg cells [9, 19].

Genome‐wide significant immune cell traits associated with IPF were extracted. Among these IPF‐associated phenotypes, those demonstrating statistically significant associations with hypothyroidism in GWAS summary statistics were further selected. Phenotypes satisfying both criteria were retained for subsequent mediation analysis.

### 2.3 Selection of IVs

To ensure the selection of appropriate IVs for the analysis, a consistent set of criteria was applied across all datasets. Genetic variants associated with hypothyroidism, IPF, and immune cell traits were identified using a stringent genome‐wide significance threshold of *p* < 1 × 10^−6^. Linkage disequilibrium pruning was performed using the PLINK software (Version 1.90), excluding SNPs with an *r* ^2^ < 0.01 in a 5000 kb window to maintain the independence of the selected variants. To further ensure the robustness of the IVs, F‐statistics for each SNP were calculated, retaining only those with an *F* value > 10. Additionally, SNPs with a minor allele frequency below 0.01 were excluded.

### 2.4 Statistical Analyses

Causal inference employed various methods, including inverse variance weighting (IVW), weighted median, MR–Egger, simple mode, and weight mode methods, all implemented via the “TwoSampleMR” R package [20]. A random‐effects model was used if the heterogeneity test indicated significant variability (*p* < 0.05). Otherwise, a fixed‐effect model was applied. Sensitivity analysis involved Cochran's *Q* test for assessing heterogeneity, MR–Egger regression for identifying pleiotropy, and the “leave‐one‐out” method for assessing the stability of individual SNP contributions. The Steiger test confirmed the direction of causality.

## 3 Results

### 3.1 Causal Relationship Between Hypothyroidism and IPF

**FIGURE 2:**
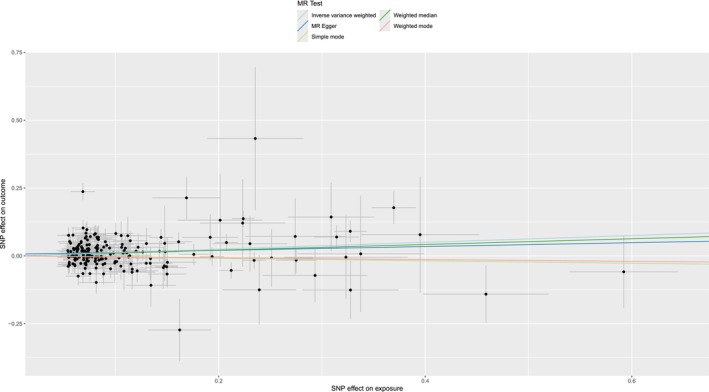
Scatterplot between hypothyroidism on idiopathic pulmonary fibrosis. MR, Mendelian randomization; SNP, single‐nucleotide polymorphism.

We selected a total of 227 index SNPs to genetically predict hypothyroidism. All selected SNPs met the minimum F‐statistic threshold (> 10), ensuring strong IVs for use in the MR analysis [21]. No overlap existed between hypothyroidism and immune cells. MR analysis using IVW indicated that hypothyroidism significantly increases the IPF risk (odds ratio [OR] = 1.13, 95% confidence interval [CI] = 1.06–1.21, *p* = 1.34 × 10^−4^) (Figure 2). Cochran's *Q* test revealed heterogeneity in this analysis (*p* < 0.05); however, as we used the random‐effects IVW method as the primary approach, this extent of heterogeneity was deemed acceptable [22]. The random‐effects model accounts for the potential variation between individual SNP estimates, providing more reliable estimates in the presence of heterogeneity [23]. No directional pleiotropy was detected by the MR–Egger intercept (*p* = 0.379). The MR Pleiotropy Residual Sum and Outlier method found no outliers (*p* = 0.175). We verified the robustness of the findings using “leave‐one‐out” analysis and funnel plots (Figures S1 and S2), ensuring no single SNP had a disproportionate effect on the outcomes. Results from other methods are provided in Table 1. Additionally, MR analysis demonstrated no reverse causality for hypothyroidism on IPF (*p* = 0.635) (Table 2).

**TABLE 1:** Results of MR analysis for hypothyroidism on IPF.

Method	*β*	SE	lo_ci	up_ci	OR	or_lci95	or_uci95	*p* value
MR–Egger	0.0705	0.0699	−0.0665	0.2075	1.0731	0.9357	1.2306	0.3140
Weighted median	0.1052	0.0445	0.0181	0.1924	1.1110	1.0182	1.2122	0.0180
Inverse variance weighted	0.1252	0.0328	0.0609	0.1895	1.1334	1.0628	1.2086	0.0001
Simple mode	−0.0451	0.1291	−0.2981	0.2080	0.9559	0.7422	1.2312	0.7280
Weighted mode	−0.0333	0.1029	−0.2350	0.1683	0.9672	0.7906	1.1833	0.7460

Abbreviations: CI, confidence interval; IPF, idiopathic pulmonary fibrosis; MR, Mendelian randomization; OR, odds ratio; SE, standard error.

**TABLE 2:** Results of MR analysis for IPF on hypothyroidism.

Method	*β*	SE	OR (95% CI)	*p*
MR–Egger	0.0469	0.0345	1.05 (0.98–1.12)	0.187
Weighted median	0.0216	0.0185	1.02 (0.99–1.06)	0.242
Inverse variance weighted	0.0067	0.0141	1.01 (0.98–1.04)	0.635
Simple mode	0.0056	0.0308	1.01 (0.95–1.07)	0.857
Weighted mode	0.0232	0.0252	1.02 (0.97–1.08)	0.366

Abbreviations: CI, confidence interval; IPF, idiopathic pulmonary fibrosis; MR, Mendelian randomization; OR, odds ratio; SE, standard error.

### 3.2 Causal Relationship Between Immune Cells and IPF

**FIGURE 3:**
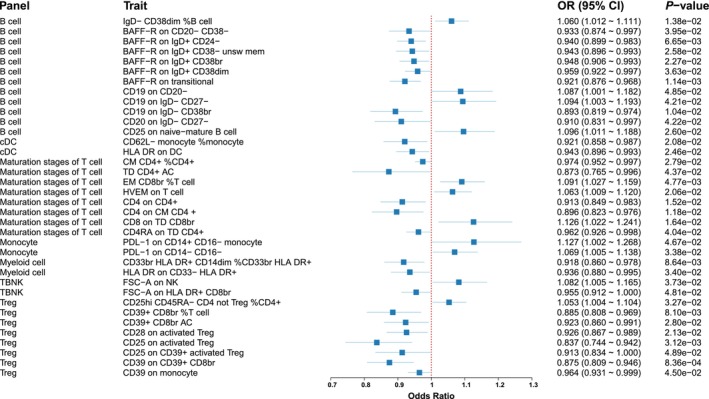
Forest plot of causal association between immune cells and idiopathic pulmonary fibrosis. OR, odds ratio; CI, confidence interval; TBNK, T cells, B cells, and natural killer cells.

We found 36 immune cell phenotypes linked with IPF, including 12 from B cells, 2 from cDCs, 8 from the maturation stages of T cells, 2 from monocytes, 2 from myeloid cells, 2 from TBNK cells, and 8 from Treg cells (Figure 3).

#### 3.2.1 B Cell Panel

**FIGURE 4:**
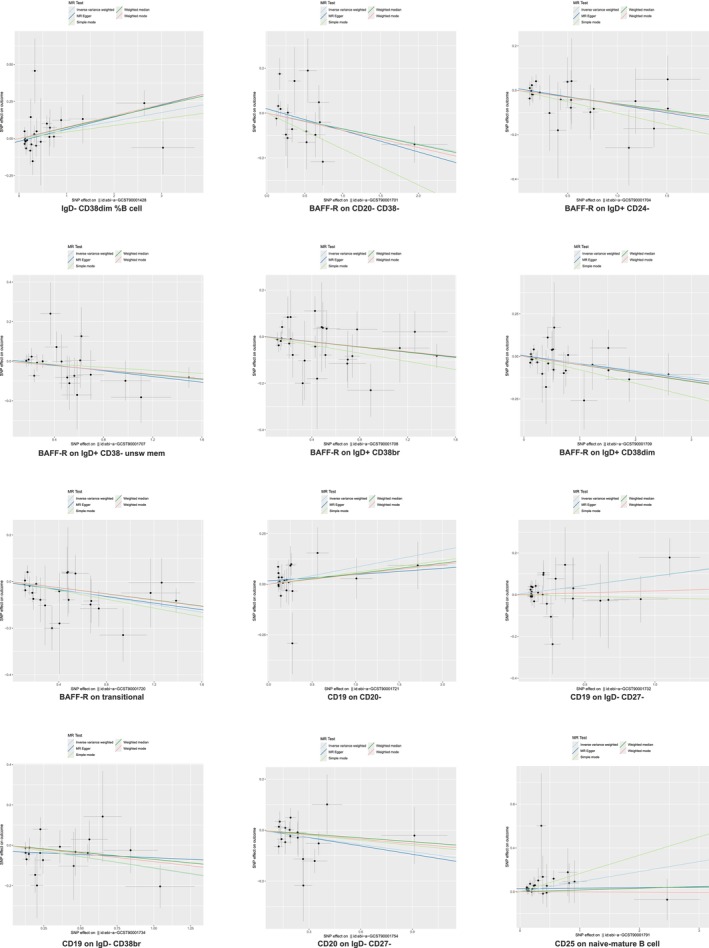
Scatter plot of the B cell panel on idiopathic pulmonary fibrosis. MR, Mendelian randomization; SNP, single‐nucleotide polymorphism.

Eight immunophenotypes demonstrated protective effects against IPF (Figure 4). Specifically, these included BAFF‐R on CD20− CD38− (OR = 0.93, 95% CI = 0.87–1.00, *p* = 0.040), BAFF‐R on IgD+ CD24− (OR = 0.94, 95% CI = 0.90–0.98, *p* = 0.007), BAFF‐R on IgD+ CD38− unsw mem (OR = 0.94, 95% CI = 0.90–0.99, *p* = 0.026), BAFF‐R on IgD+ CD38br (OR = 0.95, 95% CI = 0.91–0.99, *p* = 0.023), BAFF‐R on IgD+ CD38dim (OR = 0.96, 95% CI = 0.92–1.00, *p* = 0.036), and BAFF‐R on transitional (OR = 0.92, 95% CI = 0.88–0.97, *p* = 0.001). Conversely, four immunophenotypes were associated with increased IPF risk: IgD− CD38dim %B cell (OR = 1.06, 95% CI = 1.01–1.06, *p* = 0.014), CD19 on CD20− (OR = 1.09, 95% CI = 1.00–1.18, *p* = 0.048), CD19 on IgD− CD27− (OR = 1.09, 95% CI = 1.00–1.19, *p* = 0.042), and CD25 on naive‐mature B cell (OR = 1.10, 95% CI = 1.01–1.19, *p* = 0.026).

#### 3.2.2 cDC Panel

We identified two immunophenotypes to be associated with a reduced IPF risk (Figure S3): CD62L− monocyte %monocyte (OR = 0.92, 95% CI = 0.86–0.99, *p* = 0.021) and HLA DR on DC (OR = 0.94, 95% CI = 0.90–0.99, *p* = 0.025).

#### 3.2.3 Maturation Stages of T Cell Panel

**FIGURE 5:**
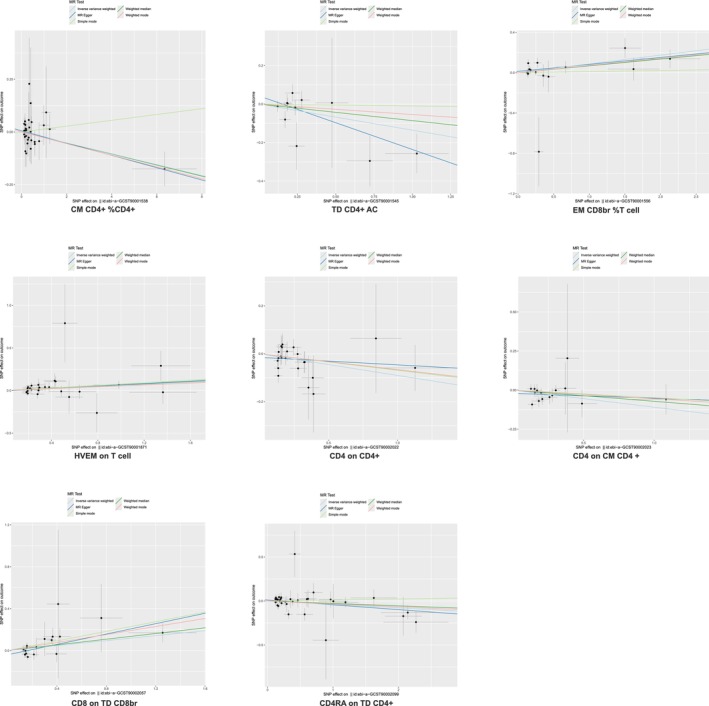
Scatter plot of the maturation stages of T cell panel on idiopathic pulmonary fibrosis. MR, Mendelian randomization; SNP, single‐nucleotide polymorphism.

Five immunophenotypes were linked to a decreased IPF risk (Figure 5): CM CD4+ %CD4+ (OR = 0.97, 95% CI = 0.95–1.00, *p* = 0.028), TD CD4+ AC (OR = 0.87, 95% CI = 0.77–1.00, *p* = 0.044), CD4 on CD4+ (OR = 0.91, 95% CI = 0.85–0.98, *p* = 0.015), CD4 on CM CD4+ (OR = 0.90, 95% CI = 0.82–0.98, *p* = 0.012), and CD4RA on TD CD4+ (OR = 0.96, 95% CI = 0.93–1.00, *p* = 0.040). Conversely, three immunophenotypes were associated with increased IPF risk: EM CD8br %T cell (OR = 1.09, 95% CI = 1.03–1.16, *p* = 0.005), HVEM on T cell (OR = 1.06, 95% CI = 1.01–1.12, *p* = 0.021), and CD8 on TD CD8br (OR = 1.13, 95% CI = 1.02–1.24, *p* = 0.016).

#### 3.2.4 Monocyte Panel

We identified two immunophenotypes associated with increased IPF risk (Figure S4): PDL‐1 on CD14+ CD16− monocyte (OR = 1.13, 95% CI = 1.00–1.27, *p* = 0.047) and PDL‐1 on CD14− CD16− (OR = 1.07, 95% CI = 1.01–1.14, *p* = 0.034).

#### 3.2.5 Myeloid Cell Panel

Two immunophenotypes were linked to a decreased IPF risk (Figure S5): CD33br HLA DR+ CD14dim %CD33br HLA DR+ (OR = 0.92, 95% CI = 0.86–0.98, *p* = 0.032) and HLA DR on CD33− HLA DR+ (OR = 0.94, 95% CI = 0.88–1.00, *p* = 0.031).

#### 3.2.6 TBNK Panel

We identified that FSC‐A on NK was associated with an increased IPF risk (OR = 1.08, 95% CI = 1.00–1.17, *p* = 0.037), whereas FSC‐A on HLA DR+ CD8br was associated with a decreased IPF risk (OR = 0.95, 95% CI = 0.91–1.00, *p* = 0.048) (Figure S6).

#### 3.2.7 Treg Panel

**FIGURE 6:**
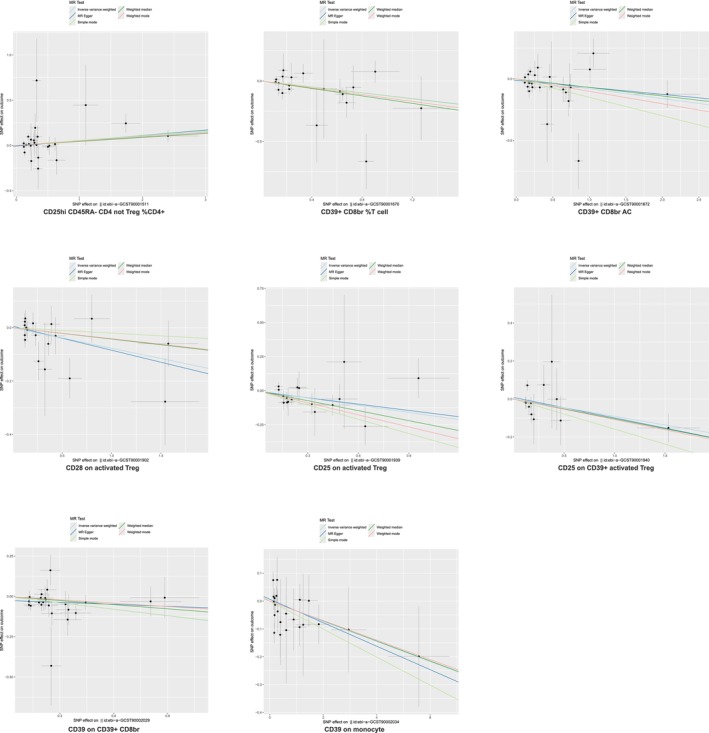
Scatter plot of the Treg panel on idiopathic pulmonary fibrosis. MR, Mendelian randomization; SNP, single‐nucleotide polymorphism.

We identified seven immunophenotypes as protective against IPF (Figure 6), including CD39+ CD8br %T cell (OR = 0.88, 95% CI = 0.81–0.97, *p* = 0.008), CD39+ CD8br AC (OR = 0.92, 95% CI = 0.86–0.99, *p* = 0.028), CD28 on activated Treg (OR = 0.93, 95% CI = 0.87–0.99, *p* = 0.021), CD25 on activated Treg (OR = 0.84, 95% CI = 0.74–0.94, *p* = 0.003), CD25 on CD39+ activated Treg (OR = 0.91, 95% CI = 0.83–1.00, *p* = 0.049), CD39 on CD39+ CD8br (OR = 0.87, 95% CI = 0.81–0.95, *p* = 0.001), and CD39 on monocyte (OR = 0.96, 95% CI = 0.93–1.00, *p* = 0.045). Only one immunophenotype, CD25hi CD45RA− CD4 not Treg %CD4+, was associated with an increased IPF risk (OR = 1.05, 95% CI = 1.00–1.10, *p* = 0.033) (Figure 6).

Additionally, we performed sensitivity analyses to evaluate heterogeneity and pleiotropy. Heterogeneity and pleiotropy tests indicated no evidence for either in the present study (*p* > 0.05). Additionally, the leave‐one‐out analysis validated the stability of the MR results. Furthermore, all Steiger test results corroborated the expected directionality of the causal relationships. Collectively, these findings indicated the consistent influence of hypothyroidism on immune cells and IPF.

### 3.3 Mediation Analysis

**FIGURE 7:**
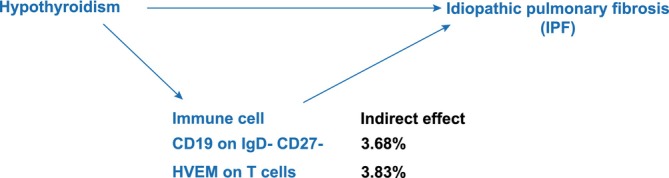
Diagram illustrating the indirect effect of hypothyroidism on idiopathic pulmonary fibrosis via immune cells.

We performed mediation analysis applying the immune cells identified as IPF mediators. We found that CD19 on IgD− CD27− and HVEM on T cells mediated the association between hypothyroidism and IPF. CD19 on IgD− CD27− was associated with an elevated risk of hypothyroidism (OR = 1.05, 95% CI = 1.00–1.10, *p* = 0.036). HVEM on T cells was similarly associated with increased levels of hypothyroidism (OR = 1.08, 95% CI = 1.00–1.16, *p* = 0.037), subsequently correlating with an elevated risk of developing IPF. Mediation analysis revealed that CD19 on IgD− CD27− accounted for approximately 3.68% of the effect of hypothyroidism on IPF, whereas HVEM on T cells mediated approximately 3.83% of this effect (Figure 7).

## 4 Discussion

IPF, a common interstitial lung disease primarily affecting older adults, is characterized by chronic progressive fibrosis and collagen deposition, resulting in worsening dyspnea, declining lung function, and a grim prognosis, frequently accompanied by a median survival period of < 5 years [24]. In this study, we conducted MR analysis to clarify the causal link between hypothyroidism and IPF. We identified the CD19 on IgD− CD27− and HVEM on T cells as critical mediators. Our findings suggested that hypothyroidism contributes to IPF pathogenesis through immune dysregulation, clarifying the causal link between hypothyroidism and IPF.

Numerous studies have explored the link between hypothyroidism and IPF [6, 7, 25]. In 2015, Oldham et al. [4] conducted a case–control study and reported for the first time that hypothyroidism is associated with a heightened risk of developing IPF and worse survival outcomes (HR = 2.12). Additionally, recent studies have employed MR to demonstrate the causal link between hypothyroidism and IPF and have shown that hypothyroidism increases the risk of developing IPF (OR: 1.12–1.14) [7, 25]. The present study similarly confirmed that hypothyroidism is a significant adverse factor for IPF, reinforcing this conclusion.

Despite the established connection, evidence regarding the mechanisms through which hypothyroidism affects IPF remains limited. Previous studies have demonstrated that mitochondrial dysfunction is a key feature of IPF [26], while evidence from animal studies has indicated that thyroid hormones may suppress pulmonary fibrosis by enhancing mitochondrial activity in lung epithelial cells [27]. Furthermore, single‐cell sequencing results indicated that thyroid hormone supplementation significantly alters gene expression in the fibrotic lung tissue of mice, modulating the crosstalk between macrophages and fibroblasts, thereby improving pulmonary fibrosis [28]. Recent studies have employed MR to elucidate the mechanisms by which hypothyroidism affects IPF, utilizing extensive plasma proteomics as an intermediary and highlighting the mediating role of the plasma protein C‐X‐C motif chemokine ligand 1 in this relationship [29].

Previous research has revealed an association between immune cell phenotypes and hypothyroidism [30], with these immune cells significantly influencing IPF pathogenesis [12]. A recent MR analysis identified 20 immune cell phenotypes associated with IPF [13], revealing some discrepancies with the present findings, where 36 immune cell phenotypes were associated with IPF. These differences may arise from the selection of GWAS databases and the criteria used for SNP filtering. Notably, in the present study, we predominantly identified MFI‐related phenotypes in B cells, which demonstrated the strongest correlation with free thyroxine levels [9]. We demonstrated a significant association between hypothyroidism and specific immune cell phenotypes implicated in IPF pathogenesis.

Our findings suggest a potential role of the immune environment, specifically CD19 on IgD− CD27− and HVEM on T cells, in mediating the link between hypothyroidism and IPF risk. Hypothyroidism increases the levels of these immune cells, thereby enhancing their profibrotic effects on IPF.

The present study had several limitations. First, the GWAS data were predominantly derived from individuals of European descent, which may restrict the broader applicability of the findings. Future research should incorporate a more diverse range of populations to improve relevance across different demographic groups. Second, the focus was solely on hypothyroidism as the exposure without further exploring the effects of thyroid hormones on immune cell phenotypes. Additionally, we observed statistically significant yet modest associations (with ORs approximately equal to 1) between hypothyroidism and specific immune cell phenotypes. These subtle effects imply limited direct mediation of IPF risk through these immune mechanisms alone, potentially reflecting minor contributions within broader disease pathways. It is important to assess the clinical relevance of these findings in conjunction with supplementary epidemiological and biological evidence. Finally, relying on summary data from GWAS instead of individual‐level data hinders a direct evaluation of the link between hypothyroidism and age‐stratified IPF. These findings require validation in large‐scale clinical trials.

In conclusion, our findings support an association between hypothyroidism and IPF risk, with observed alterations in the immune environment, particularly involving CD19 on IgD− CD27− cells and HVEM on T cells, which may play a mediating role. However, the effect sizes were modest, and limitations inherent to MR warrant caution in interpreting these findings as definitive causal pathways. Further research is needed to elucidate the underlying mechanisms. These results expand our insights into the pathophysiological association between hypothyroidism and IPF. Additional mechanistic studies are required to determine whether modulating these specific immune phenotypes (CD19 on IgD− CD27− cells and HVEM on T cells) could provide therapeutic benefits for patients with IPF.

## Author Contributions

Liang Li designed the study. Zhengling Liu and Chengkun Kou performed the research, analyzed the data, and wrote the manuscript. Xiaobo Chen, Jing Yang, Huan Zhu, Yongning Jiao, Dongyan Zhang, and Wencui Zhang analyzed the data, provided materials, and revised the manuscript.

## Ethics Statement

This study did not require separate ethical approval as it constitutes a secondary analysis of existing, published data. All primary studies included in this analysis obtained prior ethical committee approval and documented informed consent from participants.

## Conflicts of Interest

The authors declare no conflicts of interest.

## Supporting information


**Figure S1** Funnel plot of the Mendelian randomization analysis of the relationship between immune cells and idiopathic pulmonary fibrosis.

**Figure S2** “Leave‐one‐out” analysis of the Mendelian randomization association between immune cells and idiopathic pulmonary fibrosis.

**Figure S3** Scatter plot of the cDC panel on idiopathic pulmonary fibrosis. MR, Mendelian randomization; SNP, single‐nucleotide polymorphism.

**Figure S4** Scatter plot of the monocyte panel on idiopathic pulmonary fibrosis. MR, Mendelian randomization; SNP, single‐nucleotide polymorphism.

**Figure S5** Scatter plot of the myeloid cell panel on idiopathic pulmonary fibrosis. MR, Mendelian randomization; SNP, single‐nucleotide polymorphism.

**Figure S6** Scatter plot of the TBNK panel on idiopathic pulmonary fibrosis. MR, Mendelian randomization; SNP, single‐nucleotide polymorphism.

**Data S1** Supplementary information.

## Data Availability

All data were sourced from the publicly accessible GWAS database. The ethical review documents along with informed consent forms were part of the original GWAS study.
