# Supplementary figures and images for: Role of Immune Cells in Mediating the Effect of Hypothyroidism on Idiopathic Pulmonary Fibrosis
Source: Clin Respir J. 2025 Jul 10;19(7):e70111. doi: 10.1111/crj.70111 (PMC12241824; doi:10.1111/crj.70111)

MR Method

Inverse variance weighted

MR Egger

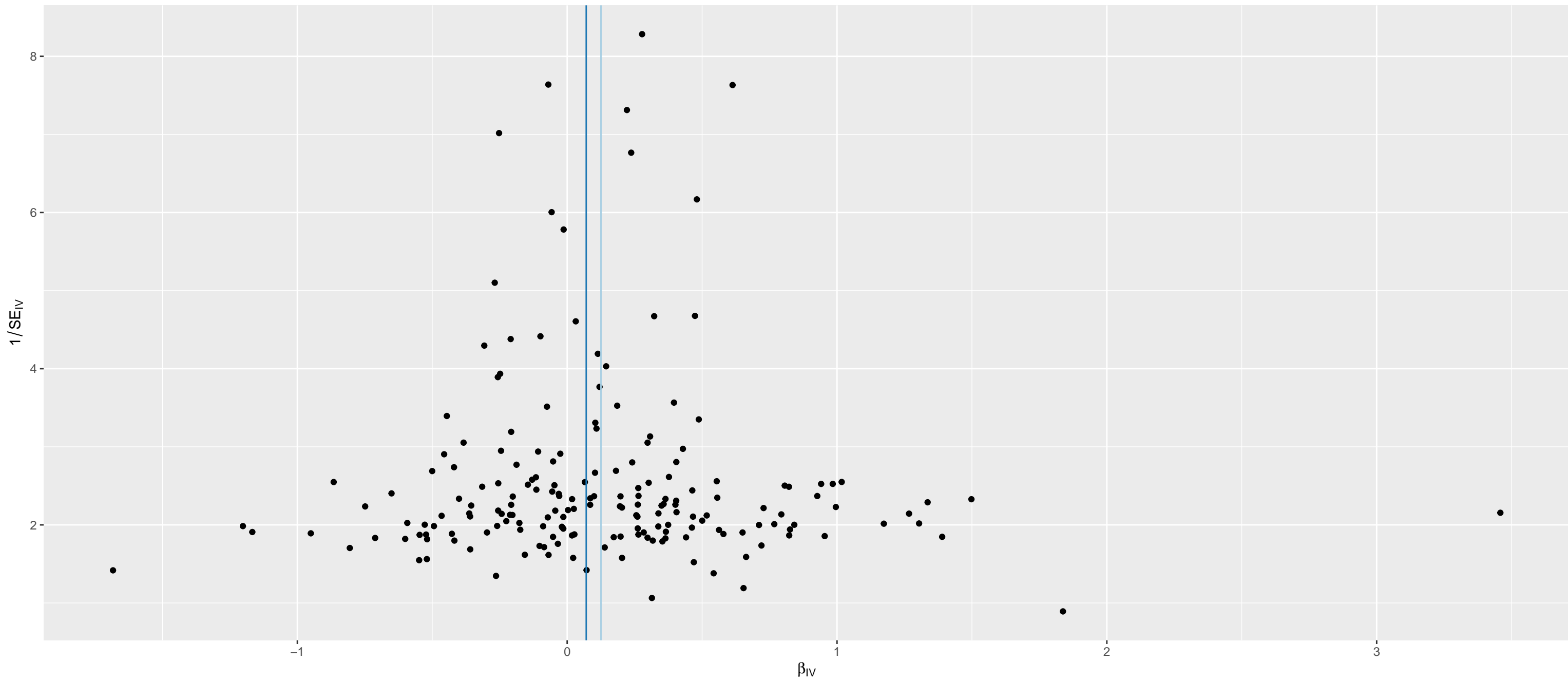

Supplement: Supplementary file 1 — Figure S1 Funnel plot of the Mendelian randomization analysis of the relationship between immune cells and idiopathic pulmonary fibrosis. [file CRJ-19-e70111-s003.pdf]

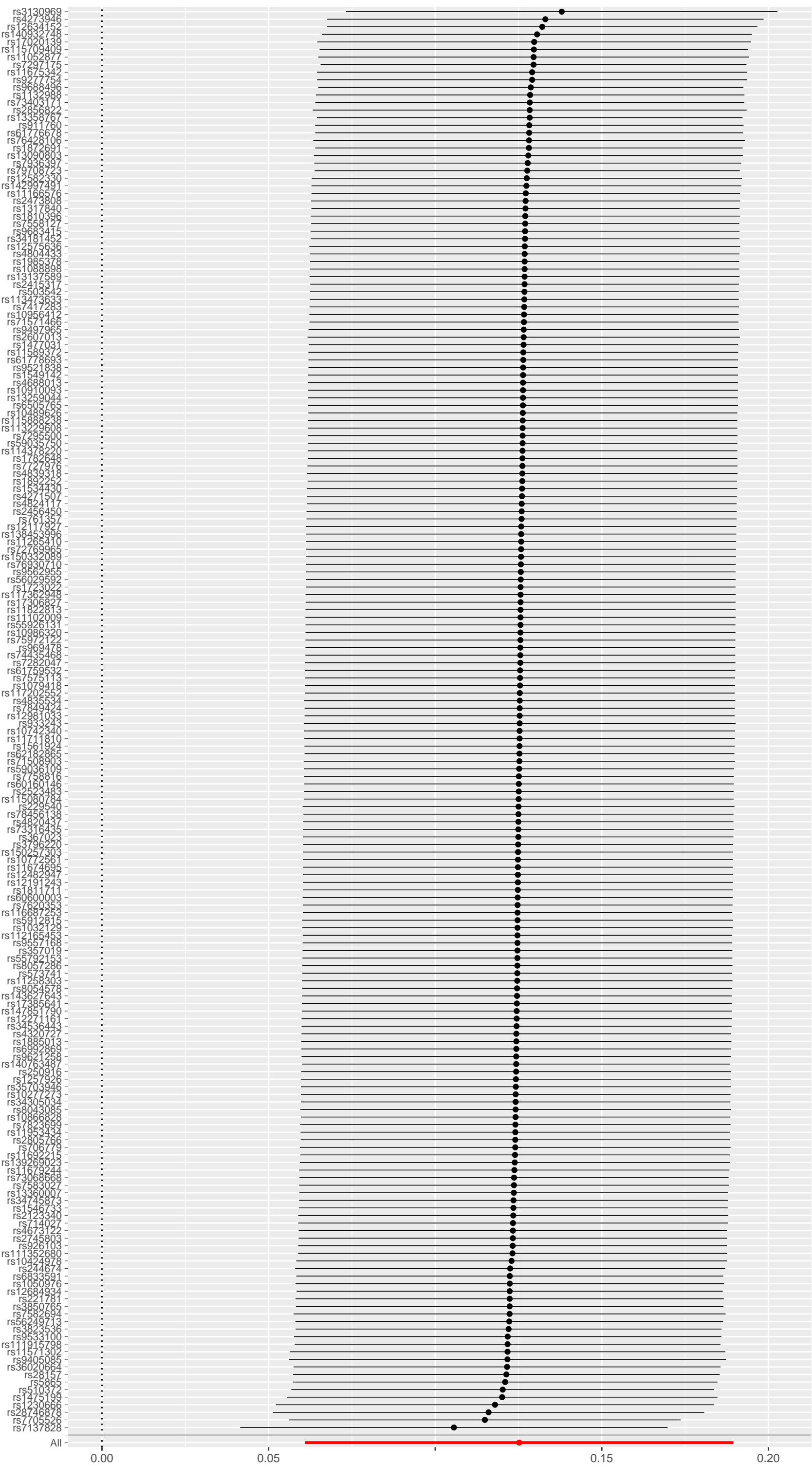

Supplement: Supplementary file 2 — Figure S2 “Leave‐one‐out” analysis of the Mendelian randomization association between immune cells and idiopathic pulmonary fibrosis. [file CRJ-19-e70111-s001.pdf]

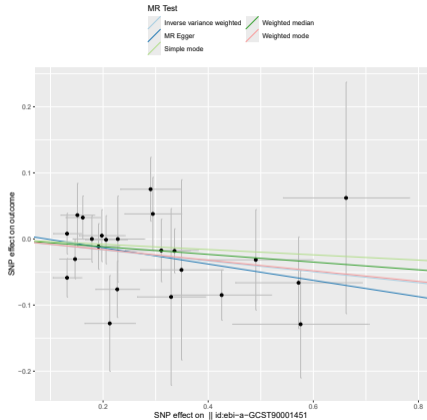

**CD62L- monocyte %monocyte**

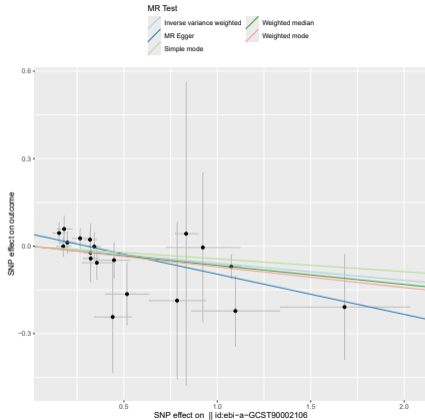

**HLA DR on DC**

Supplement: Supplementary file 3 — Figure S3 Scatter plot of the cDC panel on idiopathic pulmonary fibrosis. MR, Mendelian randomization; SNP, single‐nucleotide polymorphism. [file CRJ-19-e70111-s002.pdf]

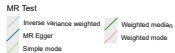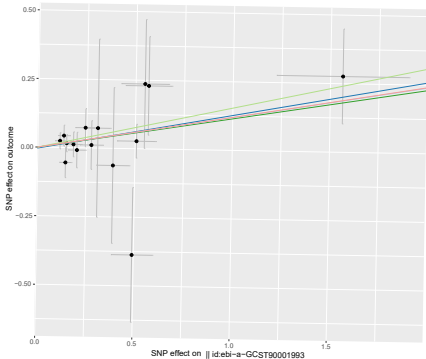

**PDL-1 on CD14+ CD16- monocyte**

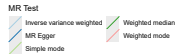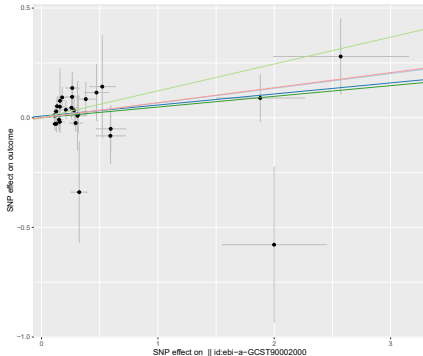

**PDL-1 on CD14- CD16-**

Supplement: Supplementary file 4 — Figure S4 Scatter plot of the monocyte panel on idiopathic pulmonary fibrosis. MR, Mendelian randomization; SNP, single‐nucleotide polymorphism. [file CRJ-19-e70111-s004.pdf]

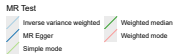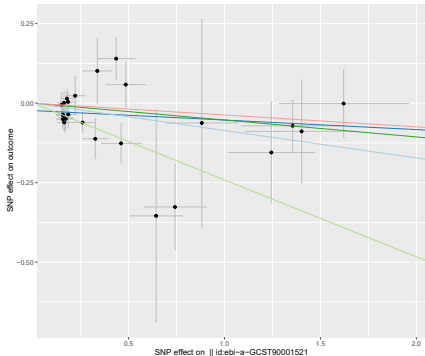

**CD33br HLA DR+ CD14dim %CD33br HLA DR+**

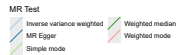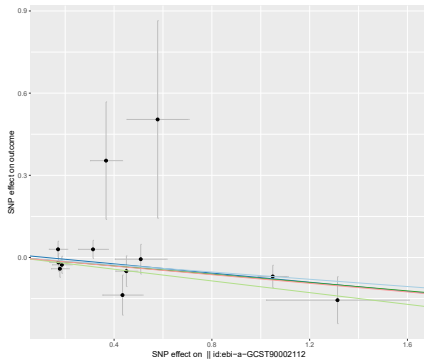

**HLA DR on CD33- HLA DR+**

Supplement: Supplementary file 5 — Figure S5 Scatter plot of the myeloid cell panel on idiopathic pulmonary fibrosis. MR, Mendelian randomization; SNP, single‐nucleotide polymorphism. [file CRJ-19-e70111-s005.pdf]

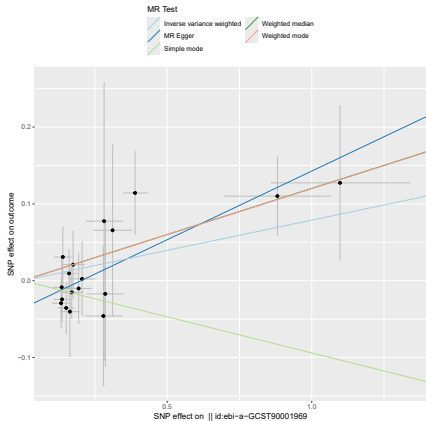

**FSC-A on NK**

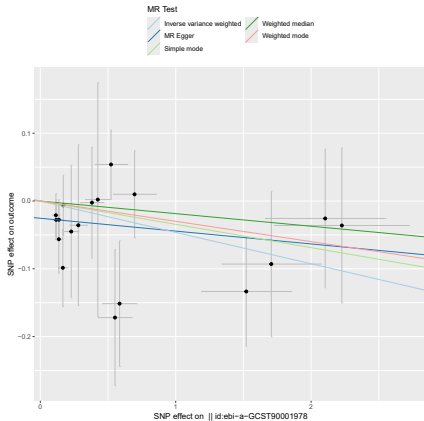

**FSC-A on HLA DR+ CD8br**

Supplement: Supplementary file 6 — Figure S6 Scatter plot of the TBNK panel on idiopathic pulmonary fibrosis. MR, Mendelian randomization; SNP, single‐nucleotide polymorphism. [file CRJ-19-e70111-s006.pdf]
