# Supplementary material for: Role of Immune Cells in Mediating the Effect of Hypothyroidism on Idiopathic Pulmonary Fibrosis
Source: Clin Respir J. 2025 Jul 10;19(7):e70111. doi: 10.1111/crj.70111 (PMC12241824; doi:10.1111/crj.70111)
Supplement: Supplementary file 7 — Data S1 Supplementary information. [file CRJ-19-e70111-s007.docx]

**Supplementary figure legends**

Figure S1. Funnel plot of the Mendelian randomization analysis of the relationship between immune cells and idiopathic pulmonary fibrosis.

Figure S2. "Leave-one-out" analysis of the Mendelian randomization association between immune cells and idiopathic pulmonary fibrosis.

Figure S3. Scatter plot of the cDC panel on idiopathic pulmonary fibrosis.

MR, Mendelian randomization; SNP, single-nucleotide polymorphism

Figure S4. Scatter plot of the monocyte panel on idiopathic pulmonary fibrosis.

MR, Mendelian randomization; SNP, single-nucleotide polymorphism

Figure S5. Scatter plot of the myeloid-cell panel on idiopathic pulmonary fibrosis.

MR, Mendelian randomization; SNP, single-nucleotide polymorphism

Figure S6. Scatter plot of the TBNK panel on idiopathic pulmonary fibrosis.

MR, Mendelian randomization; SNP, single-nucleotide polymorphism
